# Nature of Linear Spectral Properties and Fast Electronic Relaxations in Green Fluorescent Pyrrolo[3,4-c]Pyridine Derivative

**DOI:** 10.3390/ijms22115592

**Published:** 2021-05-25

**Authors:** Nataliia V. Bashmakova, Yevgeniy O. Shaydyuk, Andriy M. Dmytruk, Tomasz Świergosz, Olexiy D. Kachkovsky, Kevin D. Belfield, Mykhailo V. Bondar, Wiktor Kasprzyk

**Affiliations:** 1 Department of Experimental Physics, Taras Shevchenko National University of Kyiv, 01601 Kyiv, Ukraine; n.bashmakova@gmail.com; 2 Institute of Physics, National Academy of Sciences of Ukraine, 03028 Kyiv, Ukraine; evgs@bk.ru (Y.O.S.); admytruk@gmail.com (A.M.D.); 3 Department of Chemical Technology and Environmental Analysis, Faculty of Chemical Engineering and Technology, Cracow University of Technology, 31-155 Kraków, Poland; tomasz.swiergosz@pk.edu.pl; 4 V.P. Kukhar Institute of Bioorganic Chemistry and Petrochemistry, 02660 Kyiv, Ukraine; adkachkovsky@mail.ru; 5 Department of Chemistry and Environmental Science, College of Science and Liberal Arts, New Jersey Institute of Technology, University Heights, Newark, NJ 07102, USA; belfield@njit.edu; 6 Department of Biotechnology and Physical Chemistry, Faculty of Chemical Engineering and Technology, Cracow University of Technology, 31-155 Kraków, Poland

**Keywords:** pyrrolo[3,4-c]pyridine derivative, linear spectral properties, femtosecond transient absorption spectroscopy, quantum chemical analysis

## Abstract

The electronic nature of 4-hydroxy-1H-pyrrolo[3,4-c]pyridine-1,3,6(2H,5H)-trione (HPPT) was comprehensively investigated in liquid media at room temperature using steady-state and time-resolved femtosecond transient absorption spectroscopic techniques. The analysis of the linear photophysical and photochemical parameters of HPPT, including steady-state absorption, fluorescence and excitation anisotropy spectra, along with the lifetimes of fluorescence emission and photodecomposition quantum yields, revealed the nature of its large Stokes shift, specific changes in the permanent dipole moments under electronic excitation, weak dipole transitions with partially anisotropic character, and high photostability. Transient absorption spectra of HPPT were obtained with femtosecond resolution and no characteristic solvate relaxation processes in protic (methanol) solvent were revealed. Efficient light amplification (gain) was observed in the fluorescence spectral range of HPPT, but no super-luminescence and lasing phenomena were detected. The electronic structure of HPPT was also analyzed with quantum-chemical calculations using a DFT/B3LYP method and good agreement with experimental data was shown. The development and investigation of new pyrrolo[3,4-c]pyridine derivatives are important due to their promising fluorescent properties and potential for use in physiological applications.

## 1. Introduction

The synthesis and investigations of small fluorescent heterocyclic molecules is a subject of growing interest in the development of specific functional materials for application in a broad variety of scientific and technological areas, including organic electronics [1,2], fluorescence bioimaging [3,4], sensing [5,6], non-linear optics [7,8], tissue engineering [9], etc. Among known fluorescent heterocyclic compounds the pyrrolo[3,4-c]pyridine derivatives are promising candidates for fluorescent materials, exhibiting emission maxima in the blue–green spectral range [10], good potential for sensing [11], and probing physiological activities [12]. Features of the synthesis of this type of compound can be based on the hydrolysis and intramolecular heterocyclization of vicinal substituents in pyrid-2-one compounds [10], on the preparation of carbon dots derived from the microwave-assisted pyrolysis of citric acid in the presence of urea [13], and on the hydrolysis of alkyl 3-cyanopyridine-4-carboxylates by sulfuric acid [14]. In contrast to the majority of small heterocyclic compounds, linear spectral properties, non-linear optical parameters, and fast electronic relaxations for the molecular structure of pyrrolo[3,4-c]pyridine derivatives are scarcely addressed in scientific literature and remain subjects of intense interest.

Herein, we present linear photophysical and photochemical properties of 4-hydroxy-1H-pyrrolo[3,4-c]pyridine-1,3,6(2H,5H)-trione (HPPT), obtained previously as a fluorescent species in carbon quantum dots [13] synthesized from citric acid and urea [15], along with the comprehensive investigation of fast relaxation processes in the electronic structure of HPPT using femtosecond transient absorption spectroscopy techniques. It should be mentioned that some linear spectral data for HPPT in water were presented previously in Reference [13]. Here we continue the investigation of HPPT, including the peculiarities of excitation anisotropy, fluorescence lifetimes, and photochemical stability. The excited state absorption (ESA) spectra and characteristic vibronic relaxation times of HPPT were also obtained and characterized. The nature of electronic structure of pyrrolo[3,4-c]pyridine derivative was also analyzed by quantum-chemical calculations using TD-DFT level of theory and found to be in good agreement with experimental parameters.

## 2. Results and Discussion

### 2.1. Linear Photophysical Properties and Photostability of HPPT

**Figure 1:**
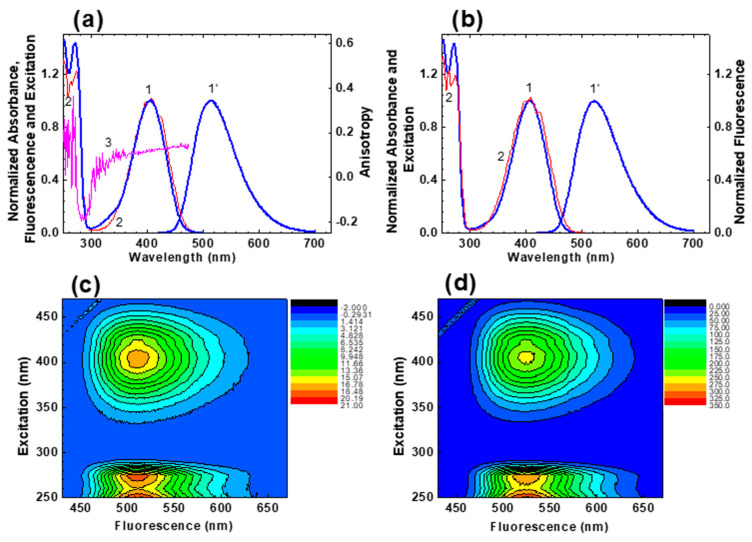
Normalized steady-state absorption (**1**), fluorescence (**1**′), and excitation (**2**) spectra of HPPT in methanol (**a**) and water (**b**). Excitation anisotropy spectrum of HPPT in glycerol ((**a**), curve 3) and 3D fluorescence maps for HPPT in methanol (**c**) and water (**d**).

The steady-state linear absorption, fluorescence, excitation, and excitation anisotropy spectra, along with the main photophysical and photochemical characteristics of HPPT, are presented in Figure 1 and Table 1, respectively.

According to these data, the linear absorption spectra were nearly independent of solvent property and exhibited no vibrational structure and relatively small extinction coefficients in the main long wavelength absorption band (maximum values, $\varepsilon^{\max}$ ~ (3.0–3.5)∙10^3^ M^−1^cm^−1^). The steady-state absorption and excitation spectra are nicely overlapped with each other (see Figure 1a,b, curves 1 and 2), and is an evidence of negligible dependence of the fluorescence quantum yield, $\Phi_{fl}$, on the excitation wavelength, $\lambda_{ex}$, and corresponding validity of the Kasha’s rule [16].

The fundamental excitation anisotropy spectrum of HPPT was obtained in viscous glycerol (Figure 1, curve 3) and characterized by relatively small ($r_{0}(\lambda )$ ≤ 0.13) and constant values in the long wavelength absorption band. These peculiarities of $r_{0}(\lambda )$ behaviour revealed a simple electronic nature of the main absorption band of HPPT that can be assigned to a single electronic transition S_0_ → S_1_ (S_0_ and S_1_ are the ground and first excited electronic state, respectively) [16], and nicely correlated with the results of quantum-chemical calculations (see Table 3 in Section 2.3). In this case the values of transition dipole moments, $\mu_{01}$, can be estimated from the experimental long wavelength absorption contour [17], with corresponding data presented in Table 1. It should be mentioned that relatively low fundamental anisotropy of HPPT in the long wavelength absorption band is most probably related to its partially anisotropic absorption transition dipole $\mu_{01}$ (i.e., $\mu_{01}^{i}\neq 0$; i = x, y, z) instead of a large angle, α, between the absorption ($\mu_{01}$) and emission ($\mu_{10}$) transition dipoles, resulting in a corresponding decrease in anisotropy as $r_{0}=(3\cos^{2}\alpha -1)/5$ [16].

**Figure 2:**
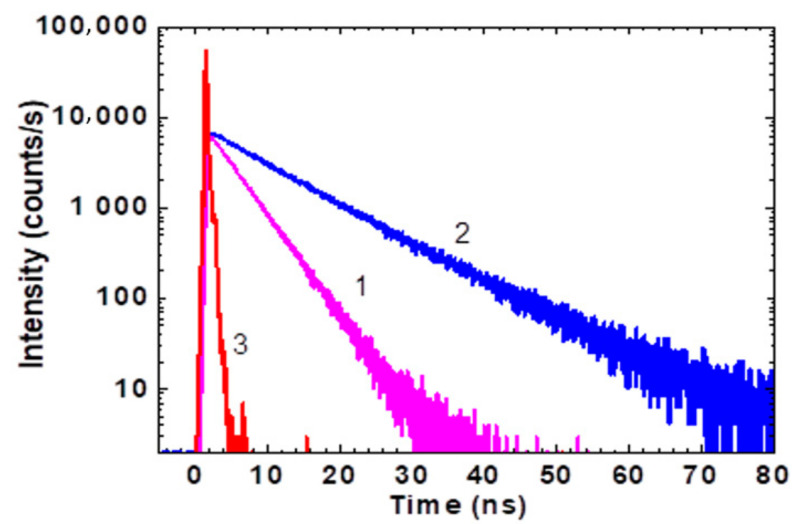
Fluorescence decay traces for HPPT in water (**1**), methanol (**2**), and instrument response function (**3**).

The steady-state fluorescence spectra of HPPT were fully independent of $\lambda_{ex}$ (see 3D fluorescence maps in Figure 1c,d), exhibited relatively large Stokes shifts (>5000 cm^−1^) and weak solvatochromic behaviour (Figure 1a,b, curves 1′). All observed fluorescence decay processes were characterized by a single exponential profile (Figure 2) with corresponding lifetimes, $\tau_{fl}$, in the range of 4–10 ns (Table 1). The values of $\tau_{fl}$ were also estimated from the experimental data based on the equation $\tau_{fl}^{cal}=\tau_{N}\cdot \Phi_{fl}$, where the natural lifetime, $\tau_{N}$, can be determined from the Strickler-Berg approach, as [18]:

(1) $$1/\tau_{N}^{}=2.9\cdot 10^{-9}\cdot n^{2}\cdot \varepsilon^{\max}\cdot \left[\int \Phi (v)dv\cdot \int [\varepsilon (v)/v]dv\right]/\left[\int [\Phi (v)/v^{3}]dv\right],$$

where n is the solvent refractive index, $\varepsilon^{\max}$ is the maximum extinction coefficient of the main long wavelength absorption band (in M^−1^·cm^−1^), Φ(v) and ε(v) are the normalized fluorescence and absorption spectra (plotted in wavenumbers, v, in cm^−1^), respectively.

**Figure 3:**
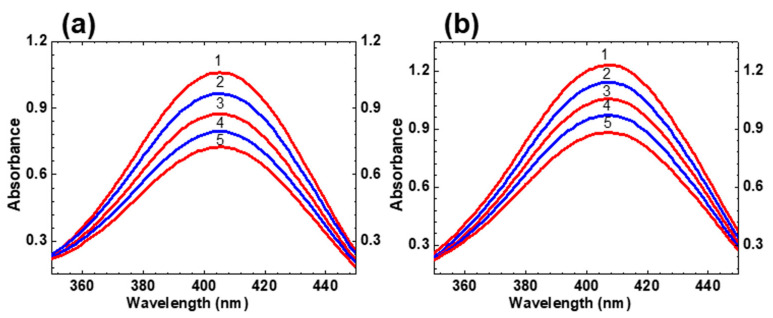
Consecutive changes in the main absorption band of HPPT in methanol (**a**) and water (**b**) under irradiation at ≈400 nm with average irradiance ≈ 40 mW/cm^2^ and irradiation times: 0 min (**1**), 4 min (**2**), 8 min (**3**), 12 min (**4**), and 16 min (**5**).

Calculated values of $\tau_{fl}^{cal}$ are presented in Table 1 and reveal sufficiently nice correlation with the corresponding experimental ones $\tau_{fl}$. That is indicative of relatively small changes in the optimized molecular geometry of HPPT under electronic excitation S_0_ → S_1_. The photostability of HPPT was estimated quantitatively by the determination of its photodecomposition quantum yield, $\Phi_{ph}$, in air-saturated methanol and water, under irradiation in the main absorption band at ≈400 nm. Typical changes in the absorption spectra after various irradiation times are presented in Figure 3.

According to these data, observed photochemical decomposition processes of HPPT can be approximately assigned to first order photoreaction [19] without essential influence of photoproducts during the first 20 min of irradiation. The values of $\Phi_{ph}$ were determined from this photodecomposition kinetics by an absorption method [20] and corresponding parameters are presented in Table 1. The highest level of photostability was observed for HPPT in water ($\Phi_{ph}$~ 5·10^−4^) and slightly decreased in methanol. It is worth mentioning that all obtained values of $\Phi_{ph}$ are comparable with the corresponding data for relatively stable laser dyes [21,22], and is important for potential practical applications of HPPT.

### 2.2. Femtosecond Transient Absorption Pump-Probe Spectroscopy of HPPT

**Figure 4:**
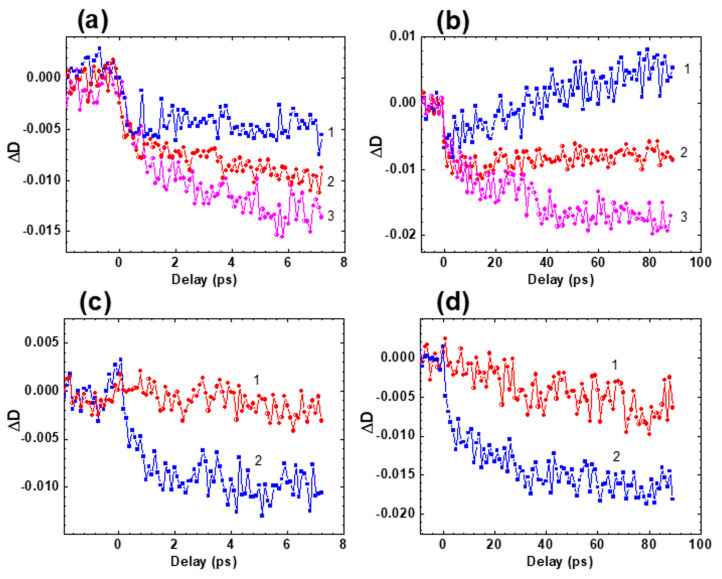
Transient absorption profiles $\Delta D=f(\tau_{D})$ for HPPT in methanol: (**a**,**b**) $\lambda_{pr}$ = 470 nm (**1**), 510 nm (**2**), 550 nm (**3**); (**c**,**d**) $\lambda_{pr}$ = 620 nm (**1**) and 560 nm (**2**).

Time-resolved transient absorption spectra and attributes of fast relaxations in the excited state of HPPT were investigated in methanol solution under femtosecond excitation in the main long wavelength absorption band at ≈ 400 nm. The dependences of the induced optical density, ΔD, on the temporal delay, $\tau_{D}$, between pump and probe pulses are presented in Figure 4 for specific probing wavelengths, $\lambda_{pr}$. Transient absorption kinetics of HPPT revealed relatively fast rise time signals (≤0.5 ps) with following dynamic changes on the picosecond time scale that, in general, can be attributed to ESA, saturable absorption (SA), and optical amplification (gain) processes [23,24]. All of the kinetic curves start with negative signals (ΔD < 0), which gradually decrease to the positive values, remain constant, or further increase depending on the spectral range (460–480 nm, 500–520 nm, and 530–620 nm, respectively). These relatively long changes are described by the characteristic times in the range of 20 to 30 ps and can be attributed to solvent reorganization phenomena, frequently observed for different organic molecules in protic solvents [25,26,27,28]. The largest values of the first emerged subpicosecond negative signals were observed in the short wavelength range ($\lambda_{pr}$ ≤ 550 nm, Figure 4a,b) and are, presumably, associated with the dominant role of optical gain from the unrelaxed first excited state S_1_ along with some possible input from the SA of the ground state S_0_. These negative signals gradually change to positive ones at $\lambda_{pr}$ ~ 470 nm in the next 20–30 ps (Figure 4b, curve 1) as evidence of substantial input of ESA processes. In contrast, further increase in the negative ΔD was observed in the spectral range $\lambda_{pr}$ ≥ 530 nm (Figure 4a,b, curves 3, Figure 4c,d), which can be explained by the increased role of optical gain.

**Figure 5:**
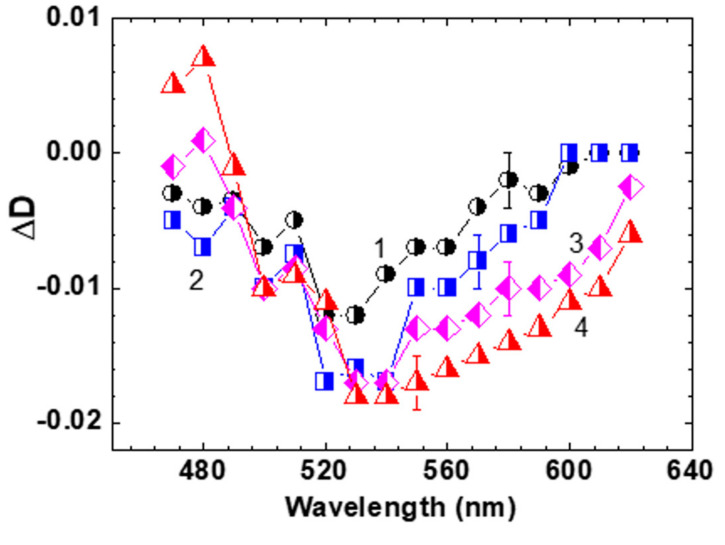
Transient absorption spectra of HPPT in methanol for $\tau_{D}$ = 1 ps (**1**), 5 ps (**2**), 30 ps (**3**), and 80 ps (**4**).

Time-resolved transient absorption spectra of HPPT (Figure 5) were obtained from the experimental dependences $\Delta D=f(\tau_{D})$ and exhibit obvious bathochromic temporal spectral shift typically observed for different types of solute-solvent interactions [26,27,29,30,31].

The spectral position of the time-resolved contour for $\tau_{D}$ ≥ 80 ps (Figure 5, curve 4) is nicely correlated with the steady-state fluorescence spectrum of HPPT (Figure 1a, curve 1′). It is worth mentioning that noticeable gain effects with maximum efficiency at ≈ 530 nm reveal some lasing potential of HPPT in liquid medium at room temperature. Nevertheless, no super-luminescence [32,33] and lasing phenomena were detected for concentrated methanol solution of HPPT under 1 kHz femtosecond excitation at ≈ 400 nm. This can be explained by the relatively high laser threshold due to the low fluorescence quantum yield and weak efficiency of radiative transitions S_1_ → S_0_, $1/\tau_{N}$ < 5 × 10^7^ s^−1^ [34].

### 2.3. Quantum Chemical Analysis of the Electronic Structure of HPPT

**Figure 6:**
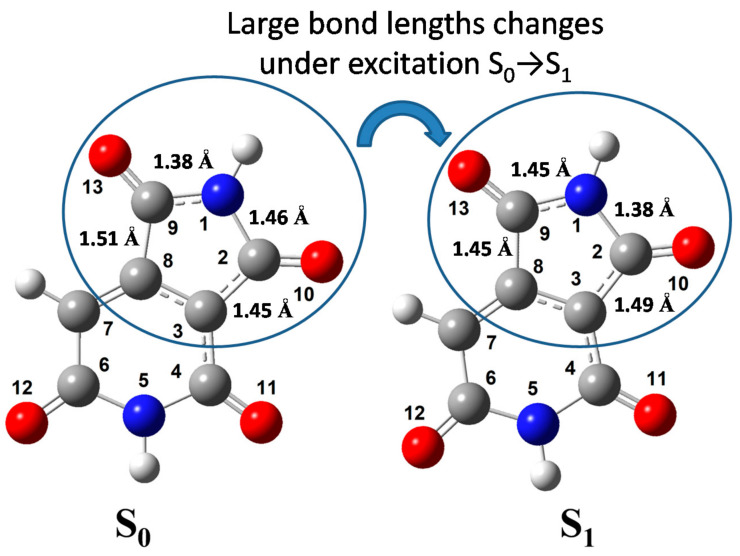
Optimized molecular geometry of HPPT in the ground (**S_0_**) and excited (**S_1_**) electronic states.

The optimization of the molecular geometry of HPPT was performed for S_0_ and S_1_ electronic states and corresponding structures are shown in Figure 6.

The values of calculated atomic charges and bond lengths are presented in Table 2, along with the corresponding changes in these values under electronic excitation S_0_ → S_1_. From the obtained data, one can see that the total negative charge is distributed dominantly at the oxygen and nitrogen atoms, while the carbon atoms bear the appreciable positive charges because of the polarization of the C-O and C-N bonds [35,36,37,38]. The electronic excitation S_0_ → S_1_ of the anionic HPPT molecule causes noticeable redistribution of the electron occupancies at both atoms and bonds. It should be mentioned that the length of four C = O bonds are minimal in both states, S_0_ and S_1_, and practically insensitive to the electronic excitation. By contrast, some C-C bonds (C2-C3, C8-C9) and C-N bonds (N1-C2, N1-C9, C4-N5) change their lengths appreciably upon excitation. It is reasonable to assume that just the changing of these bond lengths cause relatively large Stokes shift that are experimentally observed for HPPT (see Table 1).

**Figure 7:**
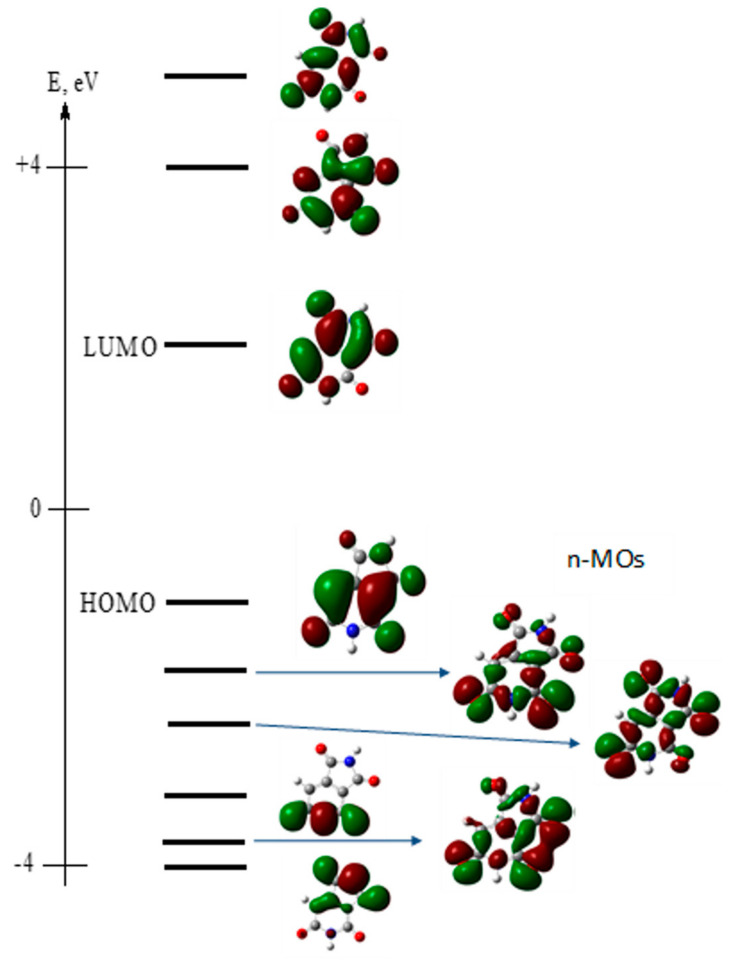
Calculated frontal and nearest molecular orbitals of HPPT.

The primary calculated electronic parameters, obtained for the optimized molecular geometry in S_0_ (for absorption) and S1 (for fluorescence) states, including frontal and nearest molecular orbitals (MOs) of HPPT, are presented in Table 3 and Figure 7, respectively.

As follows from these data, the main long wavelength absorption band of HPPT is related to a single π → π* transition (indicated as S_0_ → S_1_ and λ = 396 nm in Table 3) with relatively weak intensity (oscillator strength, f ~ 0.12) that nicely correspond to the experimentally observed absorption maxima at ~405 nm presented in Table 1. Additionally, these data correspond to the obtained constant values of excitation anisotropy in the main absorption band and small extinction coefficient, $\varepsilon^{\max}$ (see Table 1 and Figure 1a, curve 3). It should be mentioned that very weak n → π* transitions (attributed to the lone electron pair at the one-coordinated oxygen atoms) are not associated with the main absorption band and exhibit correspondingly small values of transition dipole moments. It is interesting to mention that, presumably, one of these weak transitions at 278 nm (S_0_ → S_4_, see Table 3) plays noticeable role in the excitation anisotropy spectrum (Figure 1a, curve 3), where a deep minimum is observed at ≈280 nm, which can be explained by nearly perpendicular orientation of S_0_ → S_4_ and S_0_ → S_1_ transition dipole moments. The calculated emission maximum at λ = 513 nm (see S_1_ → S_0_ transition in Table 3) and weak intensity of this transition are also in a good agreement with experimental data in Table 1. In general, the employed TD-DFT method provides a nice prediction of the absorption maxima and electronic transition dipoles of the HPPT molecule.

## 3. Experimental Section

### 3.1. Synthesis of HPPT and Linear Photophysical and Photochemical Measurements

**Figure 8:**
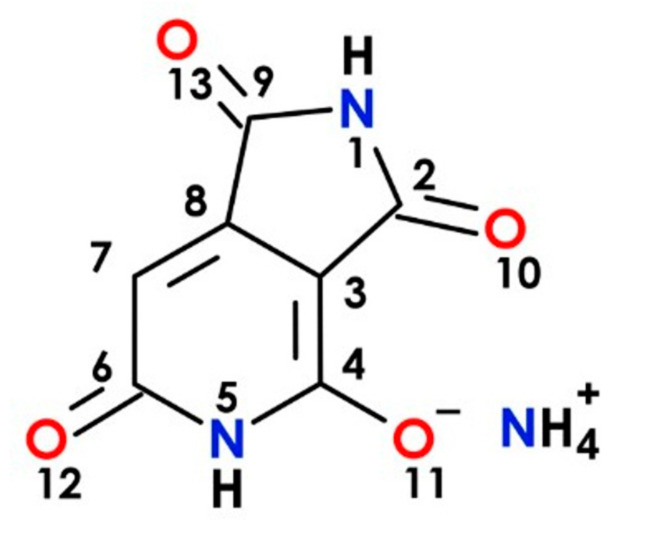
Molecular structure of the ammonium salt of HPPT.

The synthetic procedure to prepare the ammonium salt of HPPT was based on the previously reported protocol [13] with minor modifications, and the corresponding structure is presented in Figure 8. Briefly, citric acid (1.92 g, 10 mmol) was mixed with urea (6.0 g, 100 mmol) in a 15 mL glass vial and heated for 180 min at 160 °C with stirring (100 rpm). Then, the brown, solidified reaction mixture was dispersed in 250 mL of distilled water and filtered through a G4 sintered glass funnel. For the separation of HPPT, a preparative HPLC system was used (LC-20AP pumps, UV-vis SPD-20AV detector and LabSolutions 5.51 operating software, Shimadzu). The system was equipped with a preparative column C18 (250 mm × 50 mm i.d., 30 μm) (Interchim, Montluçon, France) with a 30 mm × 10 mm i.d., guard column of the same material under the following gradient system: (t [min], % A, % B, % C), (0, 100, 0, 0), (10, 100, 0, 0), (50, 0, 10, 90), (60, 0, 70, 30), (62, 0, 70, 30). The mobile phases were: A—demineralized water, B—pure acetone, C—0.4% formic acid in water. The injection volume was 30 mL and the flow rate was 50 mL/min. Detection was performed at 404 and 254 nm with a PDA UV-vis detector; and column temperature 30 °C. The HPPT fraction was collected after preparative chromatography then freeze-dried (Alpha 2–4 LO Plus, 0.37 mbar, 5 days), yielding ~200 mg of pure HPPT. The purity of the final product was confirmed via LC-DAD-ESI-MS system (LCMS-8030 Shimadzu, Kyoto, Japan). Starting materials and solvents for the synthesis and spectroscopic measurements were purchased from Sigma–Aldrich and used without further purification.

The steady-state absorption, excitation, and fluorescence spectra of HPPT were measured in air-saturated spectroscopic grade methanol and distilled water at room temperature. The steady-state absorption spectra were obtained in 1 cm path length quartz cuvettes using a Shimadzu 2450 UV-vis spectrophotometer and concentrations of sample solutions, C ~ 10^−4^ M. The steady-state fluorescence and excitation spectra were measured for dilute solutions (C ~ 10^−6^ M) in 1 cm path length spectrofluorometric quartz cuvettes using a Cary Eclipse spectrofluorometer (Varian). The acquired fluorescence spectra were corrected for the spectral responsivity of the emission detection system. The fundamental values of excitation anisotropy, $r_{0}(\lambda )$, were determined from the experimentally obtained excitation anisotropy spectrum, $r(\lambda )=r_{0}(\lambda )/(1+\tau_{fl}/\theta )$, in viscous glycerol using an “L-format” configuration ($\tau_{fl}$ and θ are the fluorescence lifetime and molecular rotational correlation time of the investigated compound, respectively) [16]. In the case of high viscosity solvent (such as glycerol at room temperature) θ >> $\tau_{fl}$ and $r(\lambda )\approx r_{0}(\lambda )$. Fluorescence lifetimes, $\tau_{fl}$, of HPPT were obtained from corresponding emission decay curves using a single photon counting technique with a Life Spec-II spectrometer (Edinburgh Instruments) and standard 1 cm path length spectrofluorimetric quartz cuvettes with low concentration solutions (C~10^−6^ M). The values of fluorescence quantum yields, $\Phi_{fl}$, were determined by relative method with 9,10-diphenylanthracene in cyclohexane as a standard ($\Phi_{fl}$ ≈ 0.95) [16]. Photochemical stability of HPPT was investigated in air-saturated methanol and distilled water under continuous wave light emitting diode irradiation with excitation wavelength $\lambda_{ex}$ ≈ 400 nm and average beam irradiance ≈40 mW/cm^2^. Corresponding values of the photodecomposition quantum yields $\Phi_{ph}=N_{mol}/N_{q}$ ($N_{mol}$ and $N_{q}$ are the number of decomposed molecules and absorbed photons, respectively) were determined by absorption methodology described previously [20].

### 3.2. Femtosecond Transient Absorption Spectral Measurements

The nature of fast intramolecular relaxation processes and time-resolved ESA spectra of HPPT were investigated by a femtosecond transient absorption pump-probe methodology described in detail previously [39,40]. In short, the output of a femtosecond laser system with 1 kHz Ti:Sapphire regenerative amplifier (Legend F-1K-HE, Coherent, Santa Clara, CA, USA) tuned to 800 nm with corresponding pulse energy, $E_{P}$ ≈ 1 mJ, and pulse duration, $\tau_{P}$ ≈ 140 fs, was divided in two parts. The first laser beam was converted into the second harmonic using 1 mm BBO crystal and employed as a pump source with excitation wavelength, $\lambda_{ex}$ ≈ 400 nm, and $E_{P}$ ≤ 5 μJ. The second part of the split output of the regenerative amplifier was attenuated and focused into a 2 mm sapphire plate to generate white-light continuum pulses, that was used as a probe source with a broad spectral range and $E_{P}$ ≤ 5 nJ. The pump and probe beams were delayed relative to each other using an optical delay line M-531.DD (PI) with a retroreflector, focused to the waists of radii ≈ 1 mm and ≈ 0.3 mm, respectively, and overlapped at a small angle in 1 mm path length quartz flow cell with the investigated solution. The transmitted through the sample cell probe beam was detected with an Acton SP2500i spectrometer coupled with a Spec 10 CCD camera (Princeton Instruments, Trenton, NJ, USA). The time resolution of this pump-probe technique was estimated as ≤300 fs.

### 3.3. Computational Analysis

All quantum-chemical calculations were performed using the Gaussian 2009 suite of programs [41]. Density functional theory (DFT) with 6–31 G(d,p) basis set and B3LYP functional were used for geometry optimization. Time dependent density functional theory (TD-DFT) was employed to describe excited states. Linear spectral properties were predicted using optimized ground and first excited singlet state molecular geometries for absorption and emission spectrum, respectively. All calculations were performed in vacuo as a simplest approach and the first step in theoretical analysis without the consideration of the solvent effects.

## 4. Conclusions

The nature of the electronic structure of HPPT molecule in liquid solutions was comprehensively studied using steady-state linear spectroscopy methods and femtosecond transient absorption pump-probe technique. Linear photophysical characterization of HPPT revealed only one electronic transition in the main long wavelength absorption band along with the origin of the large Stokes shift and relatively weak radiative transitions, as well as partially anisotropic character of transition dipoles. The photodecomposition quantum yields of HPPT were in the range of ~(5–6) 10^−4^ and are indicative of acceptable photostability for practical applications. Femtosecond transient absorption spectra of HPPT were obtained in methanol at room temperature and negative values of the induced optical density were observed over the entire fluorescence spectral range (480–600 nm), as evidence of potential super-luminescence and lasing effects. Relatively long solvate relaxation processes with the characteristic times of ≈ 20–30 ps were observed for HPPT in methanol at room temperature. The electronic structure of HPPT was also analyzed using quantum chemical calculations based on TD-DFT level of theory. The appreciable changes in charge distribution and some bond lengths of the molecular structure upon excitation were shown and were consistent with the considerably high values of the observed Stokes shifts. The majority of calculated electronic parameters of HPPT were in a good agreement with corresponding experimental data, indicative of the acceptable predictive ability of the quantum chemical methods. The steady-state and time-resolved spectroscopic investigations of pyrrolo[3,4-c]pyridine derivatives provide the impetus for further development of new citric acid-derived carbon dots, and their sensing, bioimaging and, physiological applications.

## Figures and Tables

**Table 1:** Main photophysical and photochemical parameters of HPPT in methanol and water: absorption $\lambda_{ab}^{\max}$ and fluorescence $\lambda_{fl}^{\max}$ maxima, Stokes shifts, maximum extinction coefficients, $\varepsilon^{\max}$, transition dipoles, $\mu_{01}$ (see text for details), fluorescence quantum yields, $\Phi_{fl}$, experimental $\tau_{fl}$ and calculated $\tau_{fl}^{cal}$ lifetimes, photodecomposition quantum yields, $\Phi_{ph}$.

Solvent	$\lambda_{ab}^{\max},\;\mathrm{nm}$	$\lambda_{fl}^{\max},\;\mathrm{nm}$	Stokes Shift, cm^−1^ (nm)	$\varepsilon^{\max}\;\text{×}\;10^{-3},\;M^{-1}{\mathrm{cm}}^{-1}$	$\mu_{01},\;D$	$\Phi_{fl},\;\%$	$\tau_{fl}^{cal},\;\mathrm{ns}$	$\tau_{fl},\;\mathrm{ns}$	$\Phi_{ph}$
Methanol	405 ± 1	514 ± 1	5240 (109)	4.5 ± 0.5	2.8	29 ± 2	10.7 ± 2	9.7 ± 1	6 × 10^−4^
Water	406 ± 1	523 ± 1	5510 (117)	4.0 ± 0.5	2.6	9 ± 1	3.9 ± 1	4.0 ± 0.5	5 × 10^−4^

**Table 2:** Calculated atomic charges, *q*^0^, q^*^, and bond lengths, *ι*^0^, ι^*^, for HPPT in the ground and first excited electronic state, respectively (see structure in Figure 8).

Atomic Charges and Charge Distribution upon Excitation				Bond Lengths and Their Changes upon Excitation			
Atom Number	*q* ^0^	*q* ^*^	Δ*q* = q^*^ − *q*^0^	Bond	*ι*^0^, Å	ι^*^, Å	Δ*ι* = ι^*^ − *ι*^0^, Å
1—N	−0.603	−0.583	0.020	1.2 N-C	1.456	1.3798	−0.0762
2—C	+0.509	+0.533	0.024	1.9 N-C	1.3781	1.4478	0.0697
3—C	−0.065	−0.092	−0.027	2.3 C-C	1.4461	1.4888	0.0427
4—C	+0.516	+0.533	0.017	2.10 C-O	1.2228	1.2325	0.0097
5—N	−0.605	−0.604	0.001	3.4 C-C	1.4367	1.4352	−0.0015
6—C	+0.584	+0.573	−0.011	3.8 C-C	1.4161	1.4547	0.0386
7—C	−0.265	−0.249	0.016	4.5 C-N	1.4212	1.3972	−0.0240
8—C	+0.065	+0.132	0.067	4.11 C-O	1.2335	1.2435	0.0100
9—C	+0.555	+0.424	−0.131	5.6 N-C	1.4049	1.4129	0.0080
10—O	−0.527	−0.550	−0.023	6.7 C-C	1.4505	1.4431	−0.0074
11—O	−0.563	−0.509	0.054	6.12 C-O	1.2381	1.2397	0.0016
12—O	−0.590	−0.579	0.011	7.8 C-C	1.366	1.3800	0.0140
13—O	−0.529	−0.591	−0.062	8.9 C-C	1.5062	1.4468	−0.0594
				9.13 C-O	1.2241	1.2512	0.0271

**Table 3:** Calculated electronic parameters: transition wavelengths, λ, oscillator strengths, f, transition dipoles, μ, transition types, and orbital configurations for HPPT in vacuo (HOMOs and LUMOs represent the highest occupied molecular orbitals and the lowest unoccupied molecular orbitals, correspondingly). All transitions S_0_ → S_i_ (I = 1–5) indicate absorption and transition S_1_ → S_0_ indicates fluorescence.

Transition	λ, nm	f	$\left|\mu \right|$, D	Components μ			Transition Type	Main Configuration
				${{}_{\mu }}_{x}$	${{}_{\mu }}_{y}$	${{}_{\mu }}_{z}$		
S_0_ → S_1_ Absorption	396	0.1219	3.2024	2.8835	1.3931	0.0000	π → π*	0.69 HOMO → LUMO>
S_0_ → S_2_	343	0.0001	0.0853	−0.0049	−0.0010	0.0852	n → π*	0.69 HOMO-1 → LUMO>
S_0_ → S_3_	319	0.0000	0.0110	0.0001	0.0006	−0.0110	n → π*	0.69 HOMO-2 → LUMO>
S_0_ → S_4_	278	0.0001	0.1002	−0.0004	0.0004	0.1002	n → π*	0.67 HOMO-4 → LUMO>
S_0_ → S_5_	270	0.0098	0.7497	−0.4872	−0.5699	0.0000	π → π*	0.69 HOMO-3 → LUMO>
S_1_ → S_0_ Fluorescence	513	0.0717	2.7954	−2.3859	1.4565	0.0000	π → π*	0.70 HOMO → LUMO>

## Data Availability

The data presented in this study are available on request from the corresponding authors.
